# Primary large cell neuroendocrine carcinoma of the urethra: a case report

**DOI:** 10.3389/fruro.2025.1440538

**Published:** 2025-02-06

**Authors:** Jianbai Chen, Xiaorong Mou, Zhiming Zhang, Zhiyong Nie, Jianxin Qiu

**Affiliations:** ^1^ Air Force Medical University, Xi’an, China; ^2^ Department of Pathology, Tangdu Hospital, Xi’an, China; ^3^ Department of Urology, Tangdu Hospital, Fourth Military Medical University, Xi’an, China

**Keywords:** large cell neuroendocrine carcinoma, urethra carcinoma, case report, prognosis, radical cystectomy (RC)

## Abstract

**Background:**

Primary neuroendocrine carcinoma of the urethra is a very uncommon malignant tumor, and no reports have been made about large cell neuroendocrine carcinoma (LCNEC) in the past.

**Case description:**

A 43-year-old non-smoking female patient presented with symptoms of dysuria and urination-related pain at TangDu Hospital in April 2022. A biopsy subsequently confirmed the diagnosis of primary urethral LCNEC. Following radical resection, the patient exhibited abnormal lymph node enlargement in the first month and pelvic metastases in the fourth month. Ultimately, the patient succumbed to the disease 486 days after the radical resection, attributed to widespread tumor metastases and concurrent multi-organ failure. The final pathological examination confirmed the presence of a high-grade LCNEC.

**Conclusion:**

The occurrence of primary LCNEC in the urethra is exceptionally uncommon. This particular instance was notable for its aggressive progression and unfavorable prognosis. Historically, there have been no prior documented instances of primary pure LCNEC in the urethra. It is imperative to emphasize that early identification and intervention for LCNEC could potentially offer patients a more favorable survival outcome.

## Highlights

- This is the first case of primary pure LCNEC of the urethra.
- LCNECs are aggressive and can occur within the urogenital tract in young non-smoking patients.
- The comprehensive treatment protocol and prognostic outlook for patients are described, providing a reference for the treatment of future urethral LCNEC patients.

## Introduction

Primary urethral cancer is quite rare, accounting for less than 1% of urogenital tract tumors in both men and women (1). Prior studies showed that the male-to-female ratio of urethral cancer is approximately 2.2–2.9: 1 and the average age of onset is 69.4 years old (2–4).

Neuroendocrine carcinoma (NEC) usually occurs in epithelium-containing organs, which includes carcinoid, small cell NEC, and large cell NEC. A review of the literature reveals a total of eight sporadic cases of pure NEC histology in the urethra, with three female and five male patients reported. Furthermore, prior research lacked a detailed exploration of the therapeutic approach and prognosis for patients. To the best of our knowledge, this is the first report on LCNEC in the urethra.

## Case

In April 2022, a 43-year-old female Chinese patient (height: 162 cm, weight: 52 kg) presented to our urology department with a 5-month history of progressive lower urinary tract symptoms. The patient was a non-smoker with no history of alcohol consumption and her medical history was unremarkable. The patient reported no previous history of urinary tract infections, hematuria, or pelvic surgery. She denied any weight loss, night sweats, or other systemic symptoms at the time of initial presentation. Her Eastern Cooperative Oncology Group (ECOG) performance status was 1.

**Figure 1:**
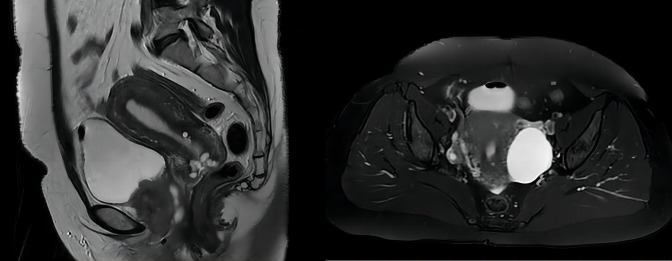
MRI scan revealed a 5.2-cm × 4.0-cm × 5.0-cm mass in the urethral tract without lymph node enlargement.

The initial presentation began with urinary frequency and urgency without apparent precipitating factors. At her first medical consultation at a local clinic, urinalysis suggested urinary tract infection, and empirical antibiotic therapy was initiated. Despite antimicrobial treatment, symptoms persisted and progressed to include dysuria and micturition-associated pain. Upon presentation at our institution, physical examination revealed tenderness on palpation in the suprapubic region and along the urethral tract. Laboratory investigations demonstrated microscopic hematuria and pyuria on urinalysis, while urine cytology was negative for malignant cells. Given the refractory nature of symptoms to conventional antibiotic therapy, a comprehensive diagnostic evaluation was undertaken. The initial MRI examination detected a 5.2-cm mass within the urethral tract, suggesting malignancy (as shown in **Figure 1**). Subsequently, an ^18^FDG PET-CT scan confirmed no metastatic or nodal disease. To definitively clarify the pathological characteristics of the lesion, an ultrasound-guided puncture biopsy was performed on 18 April 2022, confirming the presence of a malignant tumor histologically and immunologically consistent with LCNEC.

**Figure 2:**
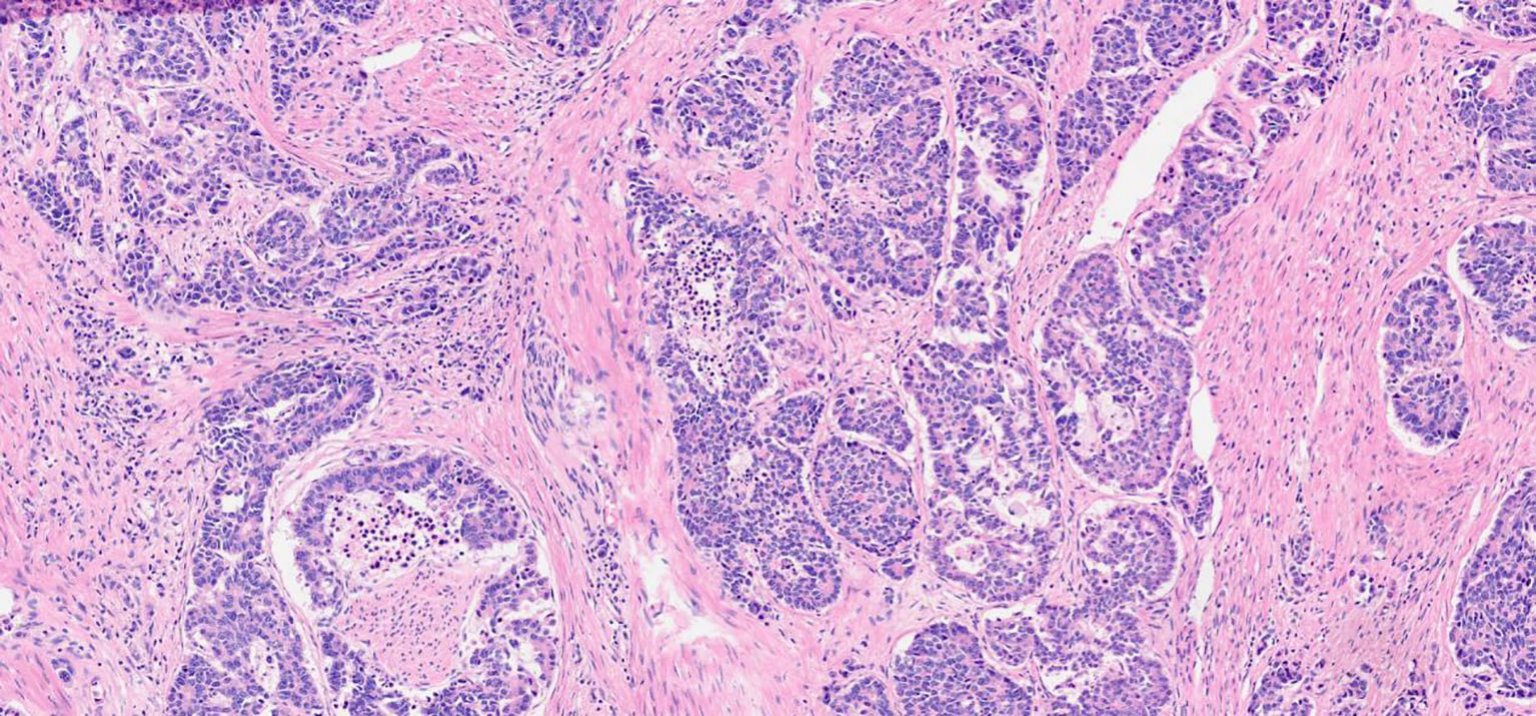
H&E-stained images of LCNEC (×100 magnification).

Given the rarity of the disease and the absence of clear treatment guidelines, a multidisciplinary team consisting of oncologists, gynecologists, urologists, and pathologists collaboratively devised the optimal treatment approach. After a thorough evaluation of the tumor size, extent of local infiltration, and review of available literature on urethral malignancies, our multidisciplinary team discussed treatment options. Given the patient’s young age (43 years) and otherwise good performance status, surgical intervention was considered. Following comprehensive counseling regarding the surgical risks, potential postoperative complications, and likelihood of tumor recurrence and metastasis, the patient expressed a strong preference for surgical management. The decision to proceed with surgery was made based on shared decision-making between the medical team and the patient. The regimen consisted of three cycles of neoadjuvant chemotherapy (cisplatin and etoposide), followed by radical cystectomy. During the neoadjuvant chemotherapy phase, a contrast-enhanced computerized tomography (CT) scan of the entire body was reassessed, revealing a stable disease status (SD). Subsequently, on 4 August 2022, the patient underwent surgery consisting of radical cystectomy, total urethrectomy, and lymph node dissection. Additionally, the urinary flow was redirected to ileovesicostomy. Pathology macroscopic examination revealed a grayish-yellow mass (4.8 cm × 4 cm × 3.2 cm) situated at the urethral tract located below the bladder, with a medium texture upon sectioning. Pathological analysis confirmed the presence of a typical large cell neuroendocrine carcinoma, infiltrating all muscle layers and surrounding connective tissue (as shown in **Figure 2**). Upon examination, positive lymphadenectasis was observed in the right pelvic region, whereas metastatic involvement was undetected in the left lymph node. Furthermore, extensive invasion of vascular/lymphatic systems and nerves was observed in the specimen, and no tumor invasion of the bladder was detected. Based on the pathological biopsy results, the patient’s tumor was staged as T2N1M0.

On 8 September 2022, the patient underwent a postoperative examination. The CT scan showed enlarged lymph nodes in the left inguinal area, approximating 0.8 cm in diameter. Consequently, our medical team administered chemotherapy consisting of cisplatin and etoposide for two cycles, along with a duration of intensity-modulated radiation therapy (IMRT). The treatment toxicity was tolerable. On 10 January 2023, a total body MRI scan confirmed the absence of enlarged lymph nodes in the left inguinal region. A total body CT scan on 25 February 2023 revealed abnormal signal intensities in the right suprapubic branch and nodular shadows in both lungs, indicating metastatic spread. With tumor progression, the chemotherapy protocol was revised to gemcitabine plus nedaplatin, with trilaciclib added to mitigate severe myelosuppression. Following two cycles of this revised treatment, the patient’s response was categorized as SD. However, grade IV myelosuppression occurred during the third cycle, reducing chemotherapy dosage by 25%. No adverse reactions were observed during the fourth cycle. A total body CT scan on 8 June 2023 showed abnormal and enlarged signals in the abdominal and pelvic regions. Nodules in both lungs were also identified, with some enlarged compared to previous imaging. Treatment response was categorized as SD based on these findings.

**Figure 3:**
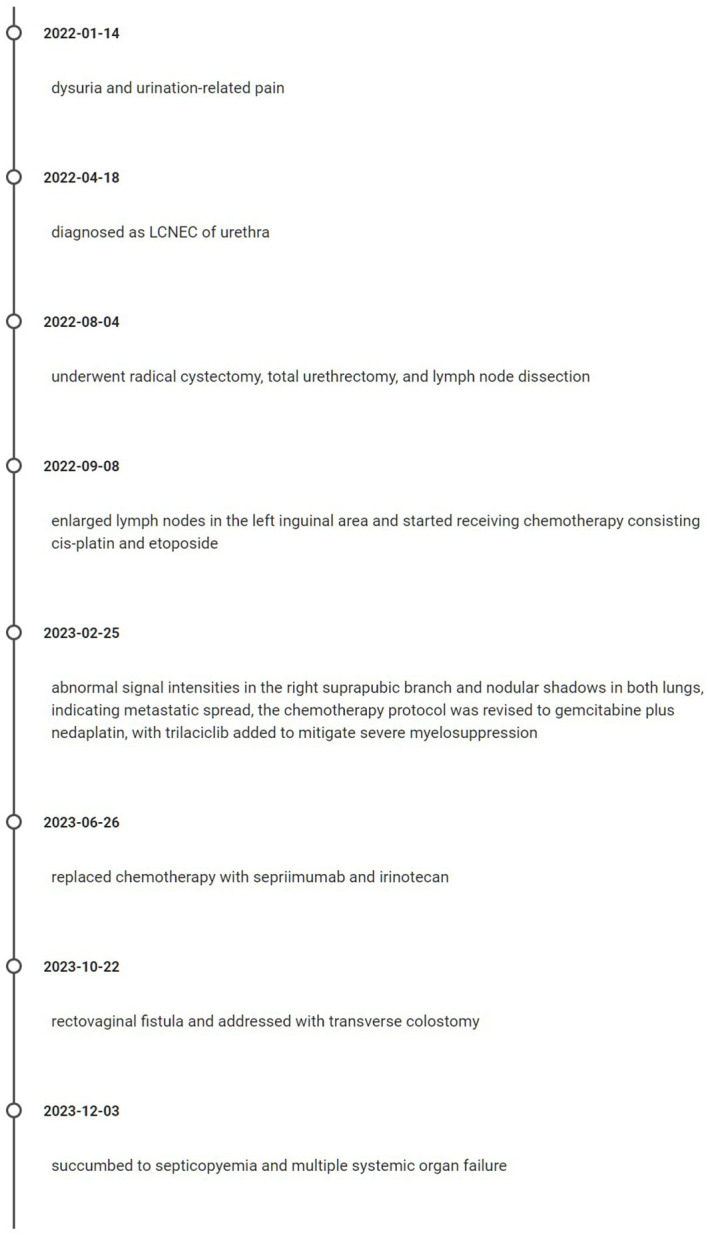
Timeline of patient's treatment and progression.

In pursuit of a better clinical outcome, the patient’s treatment protocol was changed to serplulimab plus irinotecan on 26 June 2023. No adverse reactions were noted during the treatment. Following two cycles of therapy, a total body CT scan was conducted on 3 August 2023, indicating that the multiple nodes present in both lungs had not significantly changed from prior imaging. However, a slight increase in mediastinal lymph node size was observed, as well as an enlargement of abnormal signals in abdominal and pelvic multiple nodes. Additionally, there was a notable worsening of perirectal exudate, while the lymph nodes in the right inguinal region demonstrated a reduction in size compared to previous imaging. Based on these findings, the treatment response was classified as SD. During the third cycle of chemotherapy, the patient exhibited grade III myelosuppression; therefore, the treatment regimen was adjusted to serplulimab plus lenvatinib. A total body CT scan performed on 25 September 2023 showed that multiple nodules in both lungs had reduced in size while the mediastinal lymph nodes had enlarged. Consequently, the treatment response was rated as SD. Subsequently, on 22 October 2023, the patient presented with vaginal discharge containing feces, leading to a diagnosis of rectovaginal fistula. To address this, the patient underwent transverse colostomy on 29 October 2023. However, on 18 November 2023, the patient developed chills and high fever, along with severe anemia, hypoproteinemia, urinary tract infection, and incomplete intestinal obstruction. Despite symptomatic and supportive treatment, the patient succumbed to septicopyemia and multiple systemic organ failure on 3 December 2023. The critical time points in the patient's diagnosis and treatment process are illustrated in **Figure 3**.

## Discussion

Urethral cancer patients may initially present with symptoms such as urethral obstruction, bleeding, urinary frequency, and dysuria. However, these symptoms lack specificity. Imaging plays a crucial role in the diagnosis of urethral cancer (5, 6). Ultrasonography is often the first imaging choice for urethral lesions in clinical practice, while its accuracy and sensitivity are limited (7). MRI and CT with iodinated contrast media are useful in evaluating extra-urethral extension, tumor infiltration, and lymph node metastasis in urethral cancer. However, these methods lack specificity and the diagnosis of LCNEC still relies on biopsy (8).

The optimal treatment approach for urethral LCNEC remains controversial due to its rarity. Our current strategy for treating urethral LCNEC mirrors that of lung LCNEC, with the most commonly recommended multimodality treatment consisting of surgery, chemotherapy, and radiation therapy (8). Most chemotherapy regimens are extrapolated from those used for lung LCNEC. Consequently, neoadjuvant or adjuvant combined etoposide and platinum-based therapy is theoretically preferred (9, 10). Notably, there have been no reported cases of LCNEC in the urethra. Based on the available data, the most similar case to this patient’s condition is a patient with small cell NEC. Unfortunately, these clinical data lack detailed therapeutic processes and follow-up information regarding these patients. Additionally, some of these patients had a history of other types of tumors (11–13). Most patients with NEC received a chemotherapy regimen consisting of cisplatin and etoposide, and all of them achieved some degree of efficacy. Based on the current evidence, a combination of wide surgical excision with resection of local metastases, adjuvant chemotherapy, and radiotherapy is considered the most reasonable therapeutic approach for NEC in the urethra.

Herein, we present a case where the patient experienced a recurrence and disease progression shortly following radical resection. The aggressive nature of urethral LCNEC was evident in this case, with rapid progression despite multimodal therapy. Initially, the patient’s complaints of urinary difficulty and pain during urination were not given sufficient attention. We believe that a better prognosis might have been achieved if imaging and pathological biopsy had been performed at the early stage of the disease. Early recognition of symptoms and prompt referral to specialized centers may be crucial for improving outcomes. Patient education regarding disease progression and potential complications is essential for informed decision-making throughout the treatment course. Regular quality-of-life assessments should be incorporated into the follow-up protocol. Owing to the scarcity of reported cases, the majority of research has focused on both small and large cell NECs. Consequently, there remain unresolved issues regarding the prognosis and therapeutic approach for both small and large cell NECs of the urinary tract. Future generalizations and detailed analyses of both small and large cell NECs are imperative.

Despite the unfavorable outcome, we acknowledge the patient’s and family’s trust and commitment throughout the treatment course. Their cooperation was invaluable in implementing this complex therapeutic regimen and documenting this rare clinical entity.

## Data Availability

The raw data supporting the conclusions of this article will be made available by the authors, without undue reservation.
